# Supplementary material for: Fine‐Scale Ecological Biomonitoring in a Large, Complex Agriculturally Impacted Watershed via eDNA Metabarcoding
Source: Mol Ecol. 2026 May 15;35:e70377. doi: 10.1111/mec.70377 (PMC13176897; doi:10.1111/mec.70377)
Supplement: Supplementary file 1 — Table S1: Total number of arthropod specimens collected annually by South Nation Conservation under the Ontario Benthos Biomonitoring Network (OBBN) from 2008 to 2022, with the respective number and percentage of individuals recorded as “Unidentified” at the family, genus, and species levels. While family‐level assignments were rarely unresolved, a high proportion of specimens remained unidentified at the genus and especially at the species level, highlighting limitations of morphology‐based identification. Table S3: Results of envfit analyses to test the relationship between environmental variables and community composition for three COI amplicons (BR5, F230R, MLJG). Reported values include the coefficient of determination (R 2), qualitative interpretation of effect size (very weak to very strong), and permutation p‐values (999 iterations). Significance codes: p < 0.05 (*), p < 0.01 (**), p < 0.001 (***). Figure S1: Non‐metric multidimensional scaling (NMDS) ordination plots showing macroinvertebrate community composition across land use types using DNA metabarcoding (left column) and morphology‐based identification (right column) at three taxonomic levels: family (A, B), genus (C, D), and species (E, F). Ordinations were computed using Bray‐Curtis dissimilarities, based on presence/absence data for DNA (F230R amplicons) and abundance data for morphology. DNA results are based only on 1 year of sampling (2023), while morphology results represent 10 years of sampling. Points represent individual samples, and 95% confidence ellipses group sites by land use category: mosaic landscape (green circle), predominantly agricultural (orange square), and predominantly forested (blue triangle). All morphology‐based samples are shown, including outliers. Stress values and ordination: DNA—family (0.148, k = 2), DNA—genus (0.155, k = 2), DNA—species (0.146, k = 2); Morphology—family (0.164, k = 3), Morphology—genus (0.067, k = 2), Morphology—species (0.187, k = 2). Figure S2: Tre [file MEC-35-e70377-s003.docx]

**Supplemental Information for:**

**Fine-scale ecological biomonitoring in a large, complex agriculturally impacted watershed via eDNA metabarcoding**

Bráulio S. M. L. Silva, Andrew C. Riley, Emilia Craiovan, Michael Wright, Katherine Watson, David R. Lapen, and Mehrdad Hajibabaei

| **Sampling year** | **Total specimens**  **collected** | **Unidentified Families** | **Unidentified Genus** | **Unidentified Species** |
| --- | --- | --- | --- | --- |
| 2008 | 1283 | 132 (10.29%) | 1189 (92.67%) | 1283 (100%) |
| 2009 | 228 | 7 (3.07%) | 227 (99.56%) | 228 (100%) |
| 2010 | 309 | 6 (1.94%) | 309 (100%) | 309 (100%) |
| 2013 | 9003 | 90 (1.0%) | 8935 (99.24%) | 900 (100%) |
| 2017 | 2613 | 0 | 291 (11.14%) | 2424 (92.77%) |
| 2018 | 1916 | 178 (9.29%) | 273 (14.25%) | 1752 (91.44%) |
| 2019 | 29023 | 0 | 3338 (11.5%) | 25217 (86.89%) |
| 2020 | 14350 | 0 | 2709 (18.88%) | 11383 (79.32%) |
| 2021 | 7034 | 0 | 658 (9.35%) | 5796 (82.4%) |
| 2022 | 13538 | 0 | 1274 (9.41%) | 10546 (77.9%) |

**Table S1: Total number of arthropod specimens collected annually by South Nation Conservation under the Ontario Benthos Biomonitoring Network (OBBN) from 2008 to 2022, with the respective number and percentage of individuals recorded as “Unidentified” at the family, genus, and species levels.** While family-level assignments were rarely unresolved, a high proportion of specimens remained unidentified at the genus and especially at the species level, highlighting limitations of morphology-based identification.

| **Variable** | **R²** | **R² significance** | **P value** | **Significance** | **Amplicon** |
| --- | --- | --- | --- | --- | --- |
| Water temperature (°C) | 0.138 | Moderate | 0.047 | * | BR5 |
| pH | 0.215 | Moderate | 0.006 | ** | BR5 |
| Specific conductivity (µS/cm) | 0.534 | Very Strong | 0.001 | *** | BR5 |
| Dissolved O_2_ | 0.166 | Moderate | 0.032 | * | BR5 |
| Turbidity (NTU) | 0.116 | Moderate | 0.073 |  | BR5 |
| Dissolved O_2_ (%) | 0.078 | Weak | 0.179 |  | BR5 |
| ORP (mV) | 0.099 | Weak | 0.123 |  | BR5 |
| Pressure (mmHg) | 0.122 | Moderate | 0.048 | * | BR5 |
| Water temperature (°C) | 0.333 | Strong | 0.001 | *** | F230R |
| pH | 0.273 | Moderate | 0.002 | ** | F230R |
| Specific conductivity (µS/cm) | 0.434 | Strong | 0.001 | *** | F230R |
| Dissolved O_2_ | 0.127 | Moderate | 0.073 |  | F230R |
| Turbidity (NTU) | 0.150 | Moderate | 0.043 | * | F230R |
| Dissolved O_2_ (%) | 0.037 | Very Weak | 0.462 |  | F230R |
| ORP (mV) | 0.021 | Very Weak | 0.634 |  | F230R |
| Pressure (mmHg) | 0.101 | Moderate | 0.129 |  | F230R |
| Water temperature (°C) | 0.293 | Moderate | 0.001 | *** | MLJG |
| pH | 0.375 | Strong | 0.001 | *** | MLJG |
| Specific conductivity (µS/cm) | 0.504 | Very Strong | 0.001 | *** | MLJG |
| Dissolved O_2_ | 0.100 | Moderate | 0.103 |  | MLJG |
| Turbidity (NTU) | 0.176 | Moderate | 0.024 | * | MLJG |
| Dissolved O_2_ (%) | 0.081 | Weak | 0.171 |  | MLJG |
| ORP (mV) | 0.005 | Very Weak | 0.884 |  | MLJG |
| Pressure (mmHg) | 0.253 | Moderate | 0.003 | ** | MLJG |

**Table S3: Results of envfit analyses to test the relationship between environmental variables and community composition for three COI amplicons (BR5, F230R, MLJG).** Reported values include the coefficient of determination (R²), qualitative interpretation of effect size (very weak to very strong), and permutation p-values (999 iterations). Significance codes: p < 0.05 (*), p < 0.01 (**), p < 0.001 (***).


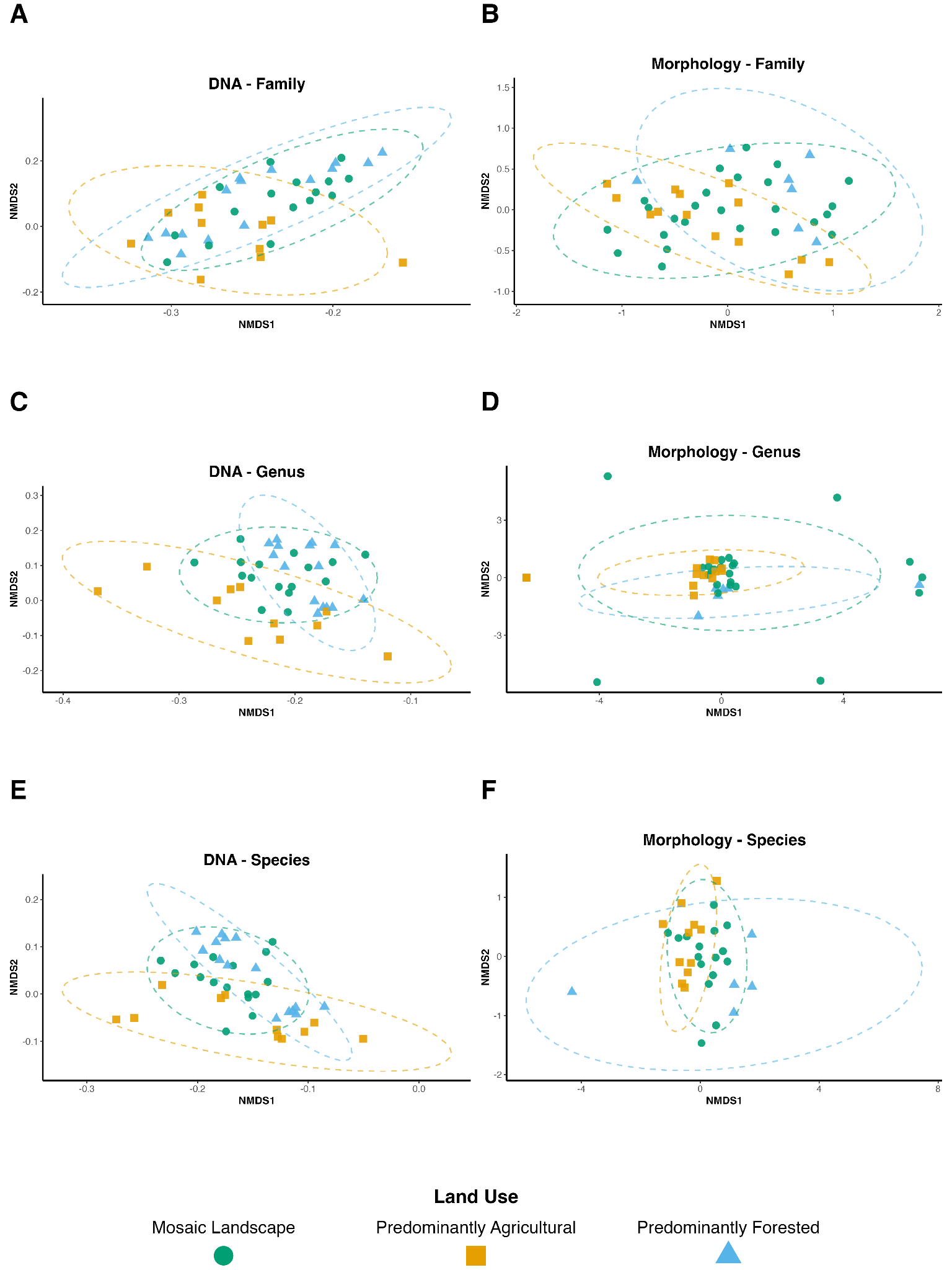


**Figure S1: Non-metric multidimensional scaling (NMDS) ordination plots showing macroinvertebrate community composition across land use types using DNA metabarcoding (left column) and morphology-based identification (right column) at three taxonomic levels: family (A, B), genus (C, D), and species (E, F).** Ordinations were computed using Bray-Curtis dissimilarities, based on presence/absence data for DNA (F230R amplicons) and abundance data for morphology. DNA results are based only on one year of sampling (2023), while morphology results represent ten years of sampling. Points represent individual samples, and 95% confidence ellipses group sites by land use category: mosaic landscape (green circle), predominantly agricultural (orange square), and predominantly forested (blue triangle). All morphology-based samples are shown, including outliers. Stress values and ordination: DNA - family (0.148, k=2), DNA - genus (0.155, k=2), DNA - species (0.146, k=2); Morphology - family (0.164, k=3), Morphology - genus (0.067, k=2), Morphology - species (0.187, k=2).


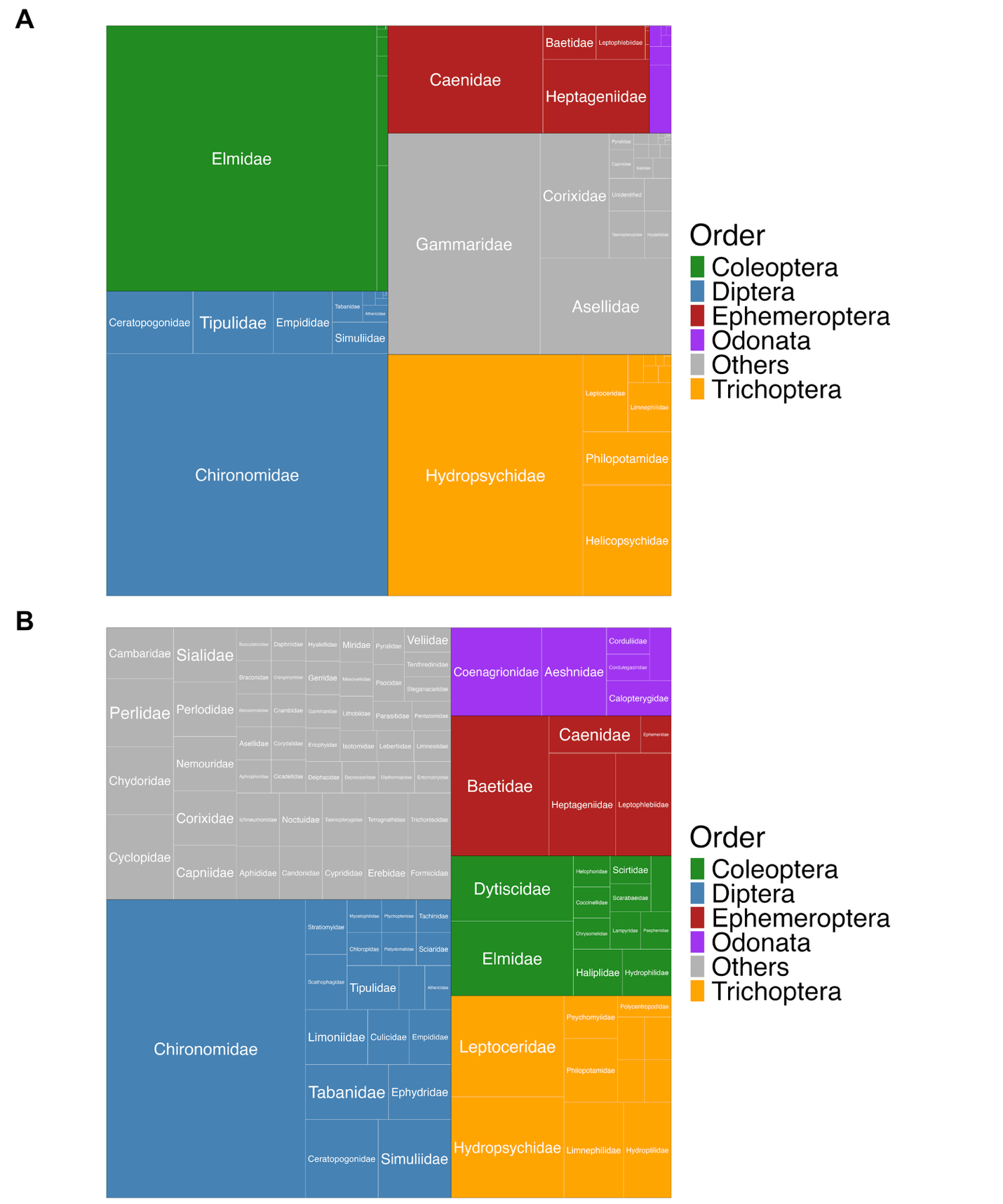


**Figure S2: Treemap visualizations of macroinvertebrate community composition based on morphology-based and DNA metabarcoding assessment methods, grouped hierarchically by order and family.** Each tile represents a family, with area proportional to (A) specimen abundance from morphological identifications or (B) the number of unique ESVs detected by DNA metabarcoding. This detailed version includes family-level labels corresponding to the order-level patterns presented in Figure 3.


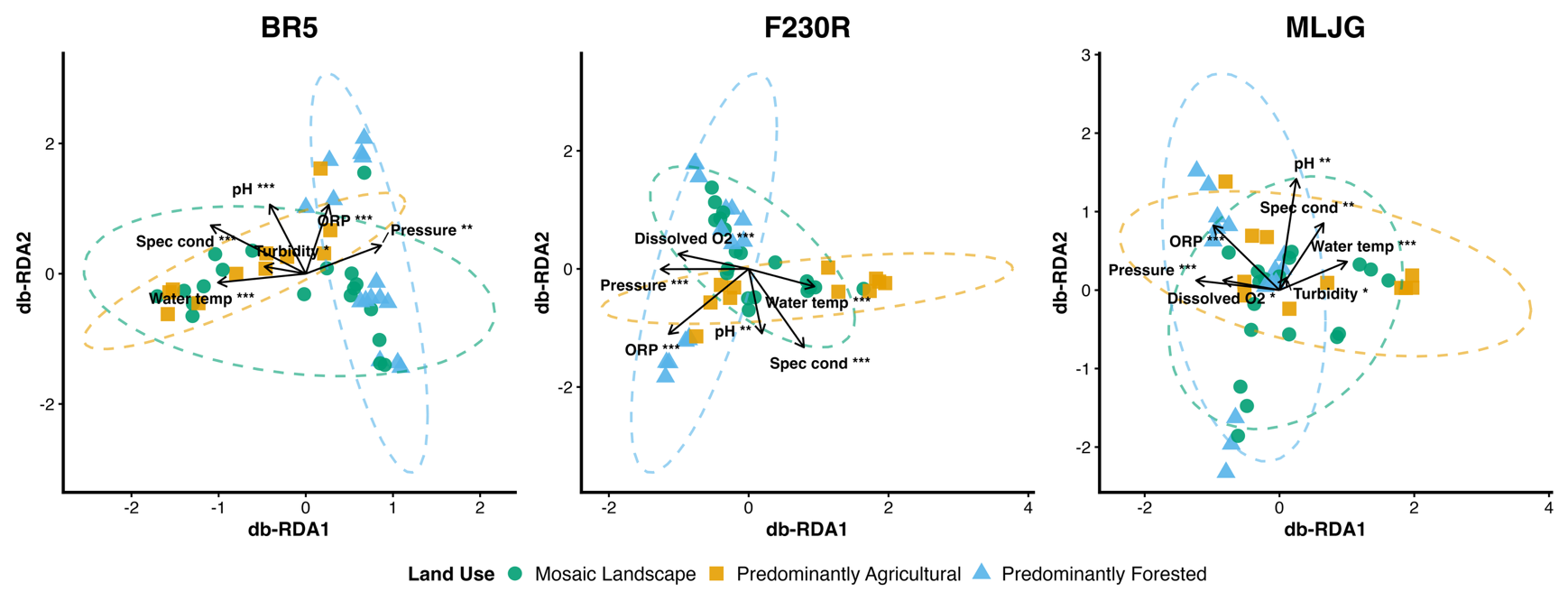


**Figure S3: Distance-based redundancy analysis (db-RDA) of macroinvertebrate community composition across land-use types.** Ordinations are based on Bray–Curtis dissimilarities and shown separately for each amplicon (BR5, F230R, and MLJG). Points represent individual replicates and are colored and shaped according to land-use category (Predominantly Agricultural, Predominantly Forested, and Mosaic Landscape). Dashed ellipses indicate 95% confidence intervals for each land-use group. Black arrows represent environmental variables, with arrow direction indicating the gradient of increasing values and arrow length proportional to the strength of the relationship with community composition. Only significant environmental variables (permutation tests, p ≤ 0.05) are displayed, with significance indicated as p ≤ 0.05 (*), ≤ 0.01 (**), and ≤ 0.001 (***). Across all amplicons, community composition was significantly associated with environmental conditions, with water temperature, pH, and specific conductivity consistently identified as the strongest predictors of variation. These results are consistent with the NMDS patterns observed in Figure 3.

| **Amplicon** | **R²** | **Adj. R²** | **ESVs** | **Replicates** | **p-value** | **F** |
| --- | --- | --- | --- | --- | --- | --- |
| BR5 | 0.312 | 0.182 | 327 | 45 | 0.001 | 2.400 |
| F230R | 0.325 | 0.198 | 635 | 45 | 0.001 | 2.555 |
| MLJG | 0.311 | 0.177 | 396 | 44 | 0.001 | 2.325 |
| **Amplicon** | **Variable** | | **Significance** | | **p-value** | **F** |
| BR5 | Water temperature (°C) | | *** | | 0.001 | 3.554 |
| BR5 | pH | | *** | | 0.001 | 2.833 |
| BR5 | Specific conductivity (µS/cm) | | *** | | 0.001 | 2.874 |
| BR5 | Dissolved O_2_ (mg/L) | | ns | | 0.074 | 1.352 |
| BR5 | Turbidity (NTU) | | * | | 0.014 | 1.678 |
| BR5 | ORP (mV) | | *** | | 0.001 | 2.518 |
| BR5 | Pressure (mmHg) | | ** | | 0.004 | 1.993 |
| F230R | Water temperature (°C) | | *** | | 0.001 | 3.143 |
| F230R | pH | | ** | | 0.001 | 2.533 |
| F230R | Specific conductivity (µS/cm) | | *** | | 0.001 | 2.711 |
| F230R | Dissolved O_2_ (mg/L) | | *** | | 0.002 | 2.081 |
| F230R | Turbidity (NTU) | | ns | | 0.075 | 1.322 |
| F230R | ORP (mV) | | *** | | 0.001 | 3.496 |
| F230R | Pressure (mmHg) | | *** | | 0.001 | 2.597 |
| MLJG | Water temperature (°C) | | *** | | 0.001 | 3.038 |
| MLJG | pH | | ** | | 0.002 | 2.421 |
| MLJG | Specific conductivity (µS/cm) | | ** | | 0.002 | 2.096 |
| MLJG | Dissolved O_2_ (mg/L) | | * | | 0.031 | 1.616 |
| MLJG | Turbidity (NTU) | | * | | 0.043 | 1.468 |
| MLJG | ORP (mV) | | *** | | 0.001 | 2.866 |
| MLJG | Pressure (mmHg) | | *** | | 0.001 | 2.766 |

**Table S6. Results of distance-based redundancy analysis (db-RDA) testing the relationship between environmental variables and macroinvertebrate community composition across three COI amplicons (BR5, F230R, MLJG).** Model summary statistics indicate that environmental variables significantly explained variation in community composition across all amplicons. Reported values include pseudo-*F* statistics and permutation-based *p*-values (999 permutations) for each environmental variable. Significance levels are indicated as p ≤ 0.05 (*), ≤ 0.01 (**), and ≤ 0.001 (***), ns (non-significant).
